# When expertise fails: detection of artificial intelligence - generated vitiligo imagery across the spectrum of dermatological experience; a cross-sectional exploratory study

**DOI:** 10.1186/s42492-026-00239-9

**Published:** 2026-11-06

**Authors:** Jonathan Shapiro, Itay Cohen, Mor Atlas, Anna Lyakhovitsky, Shany Sherman, Riad Kassem

**Affiliations:** 1 Maccabi Healthcare Services, Tel Aviv, 6812509 Israel; 2 Rutgers School of Public Health, Rutgers University, Piscataway, NJ 08901 USA; 3 Ono Academic College, Kiryat Ono, 55000 Israel; 4 Gray Faculty of Medical and Health Sciences, Tel Aviv University, Tel Aviv, 6997801 Israel; 5 Division of Dermatology, Rabin Medical Center, Beilinson Hospital, Petach Tikva, 4941492 Israel; 6 Dermatology Department, Sheba Medical Center, Ramat-Gan, 5262000 Israel

**Keywords:** Vitiligo, AI-generated image, Synthetic medical imagery, Intuitive impressions, Dermatology expertise, Expertise detection sensitivity

## Abstract

Generative artificial intelligence (AI) can produce highly realistic dermatological images, raising concerns about clinicians’ ability to distinguish authentic pathology from synthetic representations. To assess whether dermatological expertise influences the detection of AI-generated vitiligo images, and to evaluate the self-detection capabilities of the algorithm, we conducted a participant-blinded cross-sectional study in which 31 dermatologists, stratified by experience, classified one AI-generated vitiligo image alongside three real controls while blinded to the number and identity of the synthetic images. Interrater agreement was assessed using Fleiss’ kappa statistic, and the synthetic image was additionally evaluated using three large multimodal models. Residents detected the AI-generated image more frequently than senior dermatologists and key opinion leaders. After disclosure of the correct classifications, the experts identified specific biological inconsistencies, whereas residents relied primarily on intuitive impressions, and the performance of the AI models varied across the detection and post-disclosure artifact-recognition tasks. This study is limited by its evaluation of a single AI-generated image depicting a single disease (vitiligo), and the findings may therefore not be generalizable to other dermatological morphologies, image-generation prompts, or multi-image scenarios. In this exploratory study, greater clinical experience was associated with reduced detection of the evaluated synthetic image; whether this reflects a general effect of expertise requires further study. These findings highlight the emerging risks associated with medical AI and support the integration of AI-focused visual analysis into dermatology education.

## Introduction

The integration of generative artificial intelligence (AI) into biomedical imaging has progressed from experimental applications to clinical reality. AI applications in dermatology have demonstrated considerable promise for improving diagnostic accuracy, with deep learning algorithms achieving performance comparable to that of expert dermatologists in diagnosing certain skin cancers [1, 2]. Furthermore, generative AI capabilities enable the creation of synthetic medical images to address challenges such as data scarcity [3], privacy constraints, and class imbalance in rare-disease datasets [4, 5]. Researchers aim to use these tools to expand training datasets for diagnostic classifiers and to develop diverse, privacy-compliant image atlases for medical education [6–8]. A recent systematic review of generative models across multiple medical modalities found that, despite rapid technical progress, current synthesis methods often fail to incorporate patient-specific context and modality-specific clinical knowledge. Additionally, the field still lacks standardized, clinically grounded benchmarks to assess whether synthetic outputs are biologically valid rather than merely visually plausible [9]. Complementary benchmarking has demonstrated that contemporary multimodal models, while achieving strong performance on isolated visual or textual metrics, exhibit critical cross-modal disconnections and require expert-guided evaluations of anatomical and pathological plausibility. This underscores that perceptual realism is not equivalent to diagnostic fidelity [10].

Accordingly, the rapid adoption of synthetic data presents significant risks, including the underrepresentation of diverse skin tones and the generation of lesions with reduced diagnostic fidelity [11]. Although models can generate high-resolution skin textures, their diagnostic accuracy for pathological processes remains low, reaching only up to 15% [11]. Additionally, the ability of human observers to distinguish synthetic from real images has been evaluated in medical imaging, revealing that radiologists often perform no better than random guessing when viewing synthetic chest radiographs [12, 13].

This discrepancy between visual and biological realism presents a unique challenge in dermatology, which relies heavily on pattern recognition and rapid diagnostic heuristics [14–16]. In this context, distinguishing between the clinical task of diagnosing a disease and the appraisal task of identifying an AI-generated image is critical. While diagnosing a condition such as vitiligo requires clinicians to match visual findings with established pathological patterns, detecting synthetic imagery demands a critical search for digital anomalies and biological impossibilities, skills not traditionally taught in medical training.

Dermatologists rely on clinical experience to categorize findings and compare visual data with internalized diagnostic prototypes. If clinicians are susceptible to synthetic deception, then the proposed human-in-the-loop safety mechanisms for medical AI may encounter fundamental challenges [17, 18]. Therefore, this study aimed to evaluate the detectability of AI-generated vitiligo images across varying levels of dermatological expertise and to assess the self-detection accuracy of the generating algorithms.

## Methods

### Study design and participants

This participant-blinded, cross-sectional observational study recruited 31 dermatologists, who were stratified into three cohorts based on their clinical experience: 9 residents (0–4 years of training), 19 senior dermatologists (> 5 years of practice), and 3 key opinion leaders in vitiligo. These key opinion leaders were nationally recognized in this subspecialty and practiced in Israel. Participants, but not investigators, were blinded to the image provenance during the initial classification phase.

### Stimuli and biological artifact assessment

**Fig. 1:**
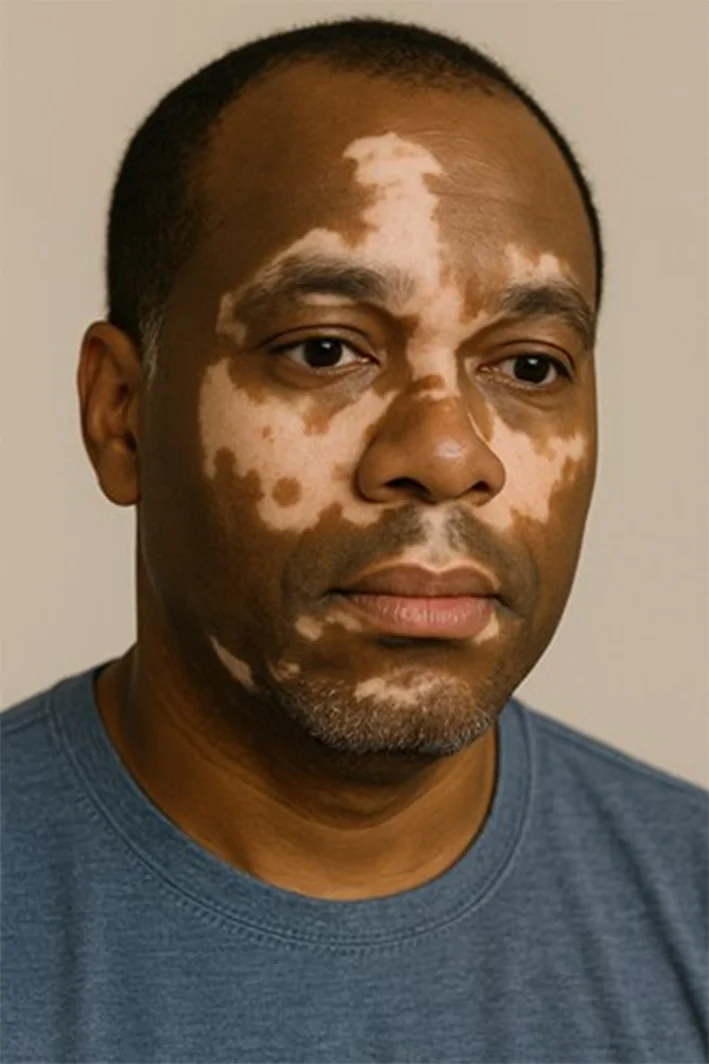
AI-generated vitiligo image

The stimuli included one synthetic image and three authentic clinical-control photographs. The synthetic image (Image 1, Fig. 1) was generated using ChatGPT-5 (OpenAI) with the prompt, “Create a real image of a patient with vitiligo on the face in different locations.” No negative prompts were applied. This image was a single, unedited, zero-shot generation: it was the first output produced for this prompt, with no batch generation or subsequent selection of a preferred image from a series. This initial output, generated without further selection, contained a dense cluster of established biological inconsistencies (detailed below), making it a stringent stimulus for evaluating comprehensive artifact recognition within a single viewing instance.

The three control stimuli (Images 2–4) were publicly available photographs of individuals with facial vitiligo, including one freely licensed stock photograph from Pexels and two images sourced from publicly accessible webpages. These images were used solely as on-screen comparison stimuli during the experiment and were not reproduced in this study.

The three source images were obtained from Pexels (https://www.pexels.com/photo/cheerful-black-man-with-vitiligo-skin-5304801/), a news feature (https://www.scmp.com/lifestyle/fashion-beauty/article/3197031/cure-vitiligo-new-us2000-treatment-restoring-skin-coloration-some-who-suffer-autoimmune-disorder), and a patient-information webpage (https://morenamia11.over-blog.es/article-diagnostico-cura-para-vitiligo-85870470.html). Control images were selected to match the synthetic image’s disease presentation, specifically facial vitiligo. All four images were displayed at comparable onscreen sizes during the experiment; therefore, the scale was not a distinguishing factor. Image resolution and lighting were not otherwise controlled, as the task focused on the biological plausibility of the depicted vitiligo pattern rather than on the image quality.

To quantify the biological inaccuracies, the synthetic image was evaluated using five established clinical parameters of vitiligo.

1. Absence of Poliosis (follicular-sparing): In true vitiligo, the destruction of melanocytes typically involves the follicular reservoir, often resulting in poliosis (hair whitening) within the depigmented patches. The AI-generated image demonstrated a “follicular-epidermal mismatch,” where the eyebrows, mustache, and beard stubble remained pigmented despite depigmentation of the underlying epidermis.
2. Geometric Pigment Borders: Natural repigmentation or depigmentation follows perifollicular or irregular geographic patterns. The AI-generated image featured a sharply demarcated, rectangular ‘block’ of pigment on the nasal sidewall, a geometric shape inconsistent with the organic, radial expansion characteristic of cutaneous disease.
3. Unnatural Linearity: Segmental vitiligo follows Blaschko’s lines, which are characteristically curvilinear. The AI-generated image displayed a rigid, ruler-straight vertical band of depigmentation extending from the forehead to the philtrum, contradicting the embryological principles of pigmentary mosaicism.
4. Commissure-sparing: In acrofacial vitiligo, the perioral region is a predilection site and typically involves the oral commissures (corners of the mouth). The AI-generated image showed perioral involvement while sparing the commissures.
5. Periocular Sparing: Acrofacial vitiligo typically presents with the “spectacle sign,” characterized by depigmentation surrounding the eyes. The AI-generated image lacked the expected periocular involvement, despite affecting the adjacent nasal and malar regions, representing a deviation from the standard acrofacial distribution pattern.

### Procedure

Participants initially classified the four images as either real or AI-generated, while remaining blinded to the prevalence of synthetic imagery. Each participant examined the images individually without receiving prior guidance from the investigators and reported their classifications to the investigators upon completion. After the disclosure of the correct classifications, the participants described, in their own words, the features indicating the image was AI-generated (post-disclosure phase, Phase II). They were not shown the five predefined features or given any response options. This open-ended, unprompted format was deliberately chosen to avoid cueing recognition and to assess whether the participants spontaneously generated these observations. Furthermore, it was intended to approximate a realistic, self-directed clinical encounter. Because the participants were recruited across several medical centers, they were divided between two investigators, with each participant assessed by a single investigator. To ensure consistency, the five feature categories and their operational definitions were prespecified and agreed upon by both investigators a priori, before data collection, and both applied the same predefined coding rules. Each participant’s spoken responses were mapped onto these predefined categories by the assessing investigator; descriptive or lay phrasing was matched to the corresponding category based on clinical meaning (for example, a remark that the eyebrows or beard remained pigmented within a depigmented patch was coded as “absence of poliosis”). Responses describing features outside the five predefined categories would have been recorded as separate, novel observations rather than being discarded; however, no participant identified features beyond the predefined framework. Responses that did not describe discrete morphological features were recorded as non-specific impressions (e.g., ‘texture,’ “digital noise,” or a general sense that the image “looked off”). The participants subsequently assessed the educational value of the experiment.

### Algorithmic benchmarking

The synthetic image was further evaluated using three large multimodal models (LMMs): ChatGPT-5.2, Gemini 3 Pro, and Claude 4.5 Sonnet, using a two-step protocol. All tests were conducted on January 24, 2026, using the standard web interfaces of the respective models. The synthetic image was uploaded to each model at a native resolution of 1024 × 1536 pixels. To account for the variable model behavior, each prompt was repeated three times per model, and the majority output was recorded. The temperature settings were maintained at the default deterministic levels where applicable. Initially, to assess baseline detection performance, the models were prompted with, *“Is this image real or AI-generated?”* Subsequently, the models were informed of the image’s synthetic nature and, to identify specific biological inconsistencies retroactively, were prompted with, *“What other clues regarding the pattern of the vitiligo in the image give a clue that it is not real?”*

### Statistical analysis

Associations between the level of expertise and correct recognition of AI-generated images were assessed using Fisher’s exact test. The prespecified primary comparison was between residents and the combined experienced cohort, which included senior dermatologists and key opinion leaders. Diagnostic performance metrics, including accuracy, sensitivity, specificity, false-positive rate, and false-negative rate, were calculated for each participant and averaged across subgroups. Each participant classified four images: one AI-generated image and three authentic control images. Sensitivity was defined as the correct identification of the AI-generated image as synthetic; specificity was defined as the correct identification of the three authentic control images as real; accuracy was defined as the percentage of all four images classified correctly; and false-negative and false-positive rates were defined as the complements of sensitivity and specificity, respectively. Because a single synthetic image was evaluated, the reported sensitivity values represented the percentage of participants in each group who correctly identified that particular image rather than the general sensitivity estimates derived from multiple synthetic cases. 95%CIs were calculated for all sensitivity and specificity estimates using the Wilson method. Interrater agreement within each group was quantified using the Fleiss’ kappa statistic. The effect size for the primary comparison was quantified using the odds ratio and phi coefficient. Because three pairwise expertise-group comparisons were possible, the significance threshold for the comparison was Bonferroni-corrected. As an additional analysis to examine physician experience as a continuous attribute, a logistic regression model was fitted to assess whether participant age, used as a proxy for clinical experience, was associated with the correct detection of an AI-generated image.

## Results

### Human detection performance

Residents demonstrated significantly higher sensitivity: 88.9% (8 out of 9; 95%​CI, 56.5–98.0) correctly identifying the AI-generated vitiligo image, compared with 36.4% (8 out of 22; 95%​CI, 19.7–57.0) of the combined experienced cohort (*P* = 0.015; odds ratio, 9.7; 95%​CI, 1.4–66.7; φ = 0.48). Overall diagnostic accuracy was 63.9% for residents, 40.8% for senior dermatologists, and 25.0% for key opinion leaders. The key opinion leader subgroup (*n* = 3) achieved 0% sensitivity, misclassifying the AI-generated image as ‘real’ in all instances.

**Fig. 2:**
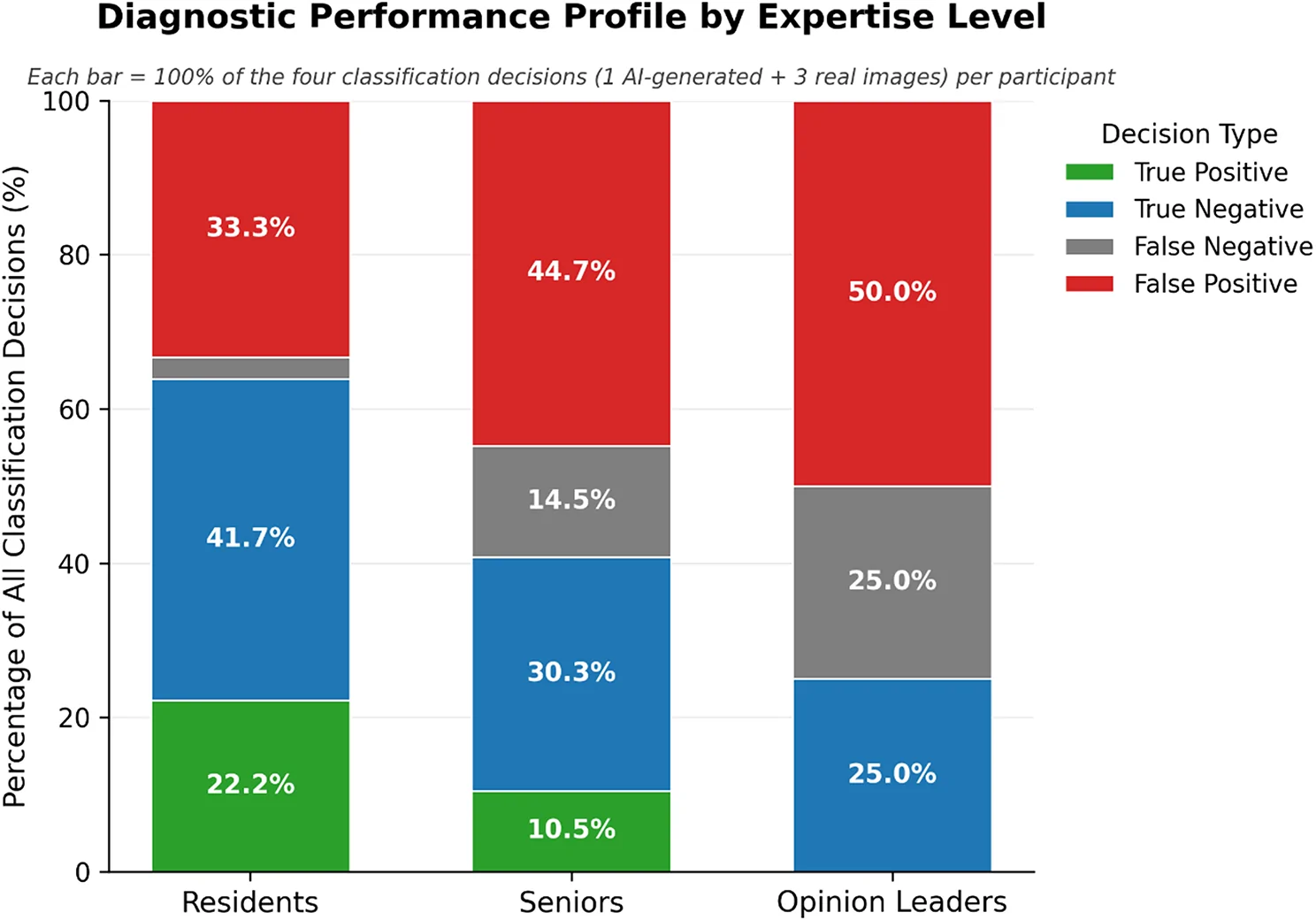
Diagnostic performance profile by expertise level. The distribution of diagnostic decisions for residents (*n* = 9), senior dermatologists (*n* = 19), and opinion leaders (*n* = 3). Each bar represents 100% of the decisions made by that group. Each colored segment is expressed as a percentage of all four classification decisions made per participant. Green (true positive): Correct identification of the AI-generated image. Note absence of the green segment in opinion leader bar representing that no opinion leader correctly identified the AI-generated image. Blue (true negative): Correct identification of real clinical photographs. Gray (false negative): Missed detection (classification of the AI-generated image as real). Note the significant gray segment for senior dermatologists and opinion leaders. Red (false positive): Incorrect classification of real images as AI-generated. The large red segment for opinion leaders indicates a high level of “confusion,” with experts frequently suspecting that real clinical images are synthetic. AI: Artificial intelligence

The complete distribution of diagnostic decisions regarding AI-generated images by expertise level is illustrated in Fig. 2, in which each bar represents 100% of the four classification decisions (one AI-generated and three real images) made per participant. Each colored segment reflects the percentage of decisions of that type, including true positives (AI-generated images correctly identified as such), false negatives (AI-generated images incorrectly classified as real), true negatives (real images correctly identified as real), and false positives (real images incorrectly classified as AI-generated).

In a logistic regression of correct detection of the AI-generated image based on participant age, the odds of detection decreased by approximately 12% per additional year (odds ratio, 0.876 per year; 95%​CI, 0.797–0.962; *P* = 0.0056).

### Interrater agreement

The key opinion leaders demonstrated perfect interrater agreement in classifying the AI-generated image (Fleiss’ kappa = 1.0, *P* = 0.0005); however, all classified it incorrectly as real. Agreement was fair among residents (κ = 0.24, *P* = 0.0039) and fair-to-moderate among senior dermatologists (κ = 0.29, *P* < 0.001).

### Post-disclosure feature recognition

**Fig. 3:**
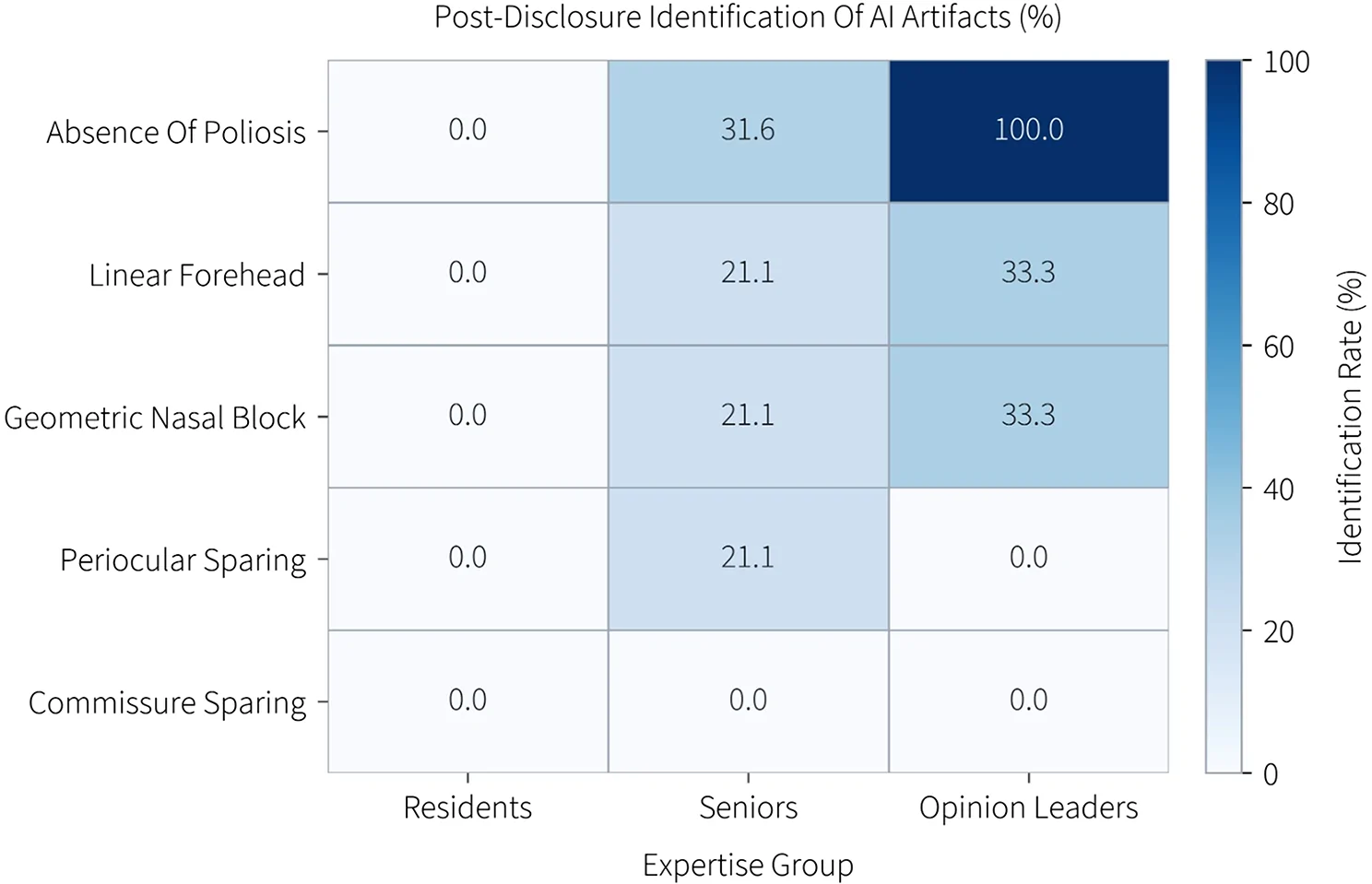
Post-disclosure identification of specific artificial intelligence artifacts. A heatmap illustrating the rate at which participants in each expertise group identified specific morphological inconsistencies after disclosure of the image’s synthetic nature. Values represent the percentage of participants in each group who correctly identified the specific feature. Darker blue indicates a higher identification rate. Residents did not identify the specific biological inconsistencies predefined in the assessment framework. By contrast, all key opinion leaders identified the absence of poliosis in the artificial intelligence-generated image and demonstrated greater recognition of geometric and linear anomalies, suggesting that their diagnostic knowledge was present but not spontaneously applied during the initial assessment. Because the key opinion leader cohort was very small (*n* = 3), the percentages presented in this heatmap are highly sensitive to individual responses. They should be interpreted as descriptive observations rather than robust statistical estimates

In the post-disclosure phase, none of the residents identified the absence of poliosis or geometric pigment borders, instead relying on the intuitive detection of texture anomalies or digital noise. In contrast, all key opinion leaders (3 out of 3) identified the absence of poliosis (Fig. 3). Furthermore, 33.3% of the key opinion leaders (1 out of 3) identified subtle geometric artifacts, including the rectangular pigment block and linear forehead strip.

### LMM performance

The model used to create the image, ChatGPT-5, classified it as ‘real.’ The updated version, ChatGPT-5.2, correctly identified the AI-generated image but incorrectly flagged one authentic clinical photograph as AI-generated. During post-disclosure feature extraction, ChatGPT-5.2 correctly identified 4 out of 5 biological inconsistencies. Claude 4.5 Sonnet initially failed to detect the AI-generated image, classifying it as real; however, in the post-disclosure phase, it identified 100% of morphological inconsistencies (5 out of 5). Gemini 3 Pro successfully detected the AI-generated image at baseline but exhibited a high false-positive rate, incorrectly classifying 66% of the authentic photographs as AI-generated. It also identified all five morphological inconsistencies in the post-disclosure phase.

## Discussion

In this exploratory study, residents identified the selected AI-generated vitiligo image more frequently than experienced dermatologists. Because a single image was evaluated, this finding should be interpreted as specific to the selected image and study setting rather than as evidence that greater expertise generally reduces the detection of synthetic dermatological images. Further studies using larger and more diverse image sets are needed to determine whether this pattern generalizes.

However, in the post-disclosure phase (Phase II), residents struggled to articulate specific biological inconsistencies, whereas key opinion leaders readily identified definitive biologically implausible features such as the absence of poliosis. This gap suggests that the expert knowledge required to detect the synthetic image was present but may not have been spontaneously applied during the initial blinded assessment. Detecting a synthetic image is a task distinct from diagnosing the depicted disease; the participating experts reliably recognized the vitiligo presentation yet did not engage in the separate critical appraisal required to identify the image’s artificial origin.

These findings are consistent with the cognitive theories of pattern recognition in medical diagnosis [19–23]. Because cognitive processing was not directly measured, the following interpretation should be viewed as hypothesis generation rather than an established mechanism: dermatological training may condition experts to rely heavily on rapid, heuristic-based processing (System 1 thinking) when identifying pathologies [14, 24–28]. The high degree of global realism of the synthetic image may have activated this rapid pattern recognition in experts, prompting them to focus on the vitiligo presentation rather than critically evaluating whether the image was synthetic.

In contrast, residents, who may have less rigid diagnostic templates, may have been more likely to engage in deliberate, analytical processing (System 2 thinking), enabling them to detect pixel-level incongruences. Because clinical experience and clinician age were closely linked in this sample, the possibility that age-related perceptual factors or differing familiarity with digital imagery, rather than clinical expertise alone, contributed to these differences cannot be excluded. Consequently, established clinical heuristics may inadvertently reduce sensitivity to digital anomalies [14, 24, 28–30].

The LMM benchmarking revealed a fragmented landscape of algorithmic reliability. The model behaviors mirrored the human cognitive spectrum; some models prioritized high-level semantic coherence and failed at initial detection, whereas others demonstrated high sensitivity but low specificity, leading to false-positive classification of authentic images.

A false-positive rate exceeding 33% represents a substantial barrier to clinical integration, because automated screening tools could erroneously flag legitimate patient records as fraudulent. Furthermore, the limited ability of the generator model to consistently recognize its output represents a potential risk of model collapse, whereby future AI systems trained on data containing undetectable synthetic images may experience performance degradation [31–34]. In dermatology, the contamination of diagnostic datasets with synthetic images could significantly impact future algorithmic performance [11, 35].

Although high-fidelity synthetic data offer potential solutions to challenges, such as privacy constraints and data scarcity [36–38], these data introduce significant risks, including the potential for telemedicine fraud [39]. Current pedagogical models, which rely heavily on pattern recognition, may require adaptation to the era of generative AI [14, 24, 25].

Therefore, medical education should transition toward incorporating AI literacy and training in the identification of generative biomarkers, specific artifacts indicative of algorithmic origins, such as logical inconsistencies or geometric artifacts [40–45]. As evidenced by the participants’ high educational value ratings, the critical evaluation of synthetic images encourages clinicians to engage in metacognition, prompting a more analytical approach that may enhance overall diagnostic reasoning.

## Limitations

This study had several limitations. It relied on a single AI-generated image depicting the dermatological condition vitiligo. Although this zero-shot synthetic image contains multiple distinct biological artifacts within a single image, its high global realism may have overridden expert scrutiny during the initial blinded assessment. Therefore, the findings should not be assumed to generalize to other AI-generated dermatological images, disease morphologies, or image-generation prompts in which the balance between global realism and detectable biological artifacts may differ. This exploratory benchmark study provides a foundation for future multi-image investigations.

The key opinion leader subgroup was small (*n* = 3), which might have affected the reliability of the subgroup-level estimates. Post-disclosure Phase II feature-identification analysis was intentionally performed after participants were informed that the image was synthetic; therefore, it should be interpreted as a measure of post-disclosure artifact recognition rather than detection ability under blinded conditions. Because all participants were exposed to the same disclosure, this design did not preclude descriptive comparisons between the expert groups. Clinician age and clinical experience were strongly collinear in this cohort, as residents were uniformly younger than experienced clinicians. Consequently, the design could not disentangle the contribution of chronological age from that of clinical experience, and alternative explanations such as age-related perceptual differences or greater familiarity with digital imagery among younger clinicians cannot be excluded. This study used only three authentic control images; therefore, the findings depend on the specific stimuli that were selected. The expert groups were also unequal in size. Participant confidence ratings were not collected, which precluded the analysis of the relationship between certainty and accuracy. Post-disclosure responses were open-ended and coded by a single investigator who administered each assessment using shared, predefined criteria. Because raw responses were not retained for re-coding, formal interrater reliability was not assessed. The algorithmic benchmarking was limited in scope, given the small number of runs per model, and should be regarded as a preliminary exploratory assessment rather than a comprehensive evaluation of model performance. Finally, the real-world detection rates in a high-volume clinical workflow may be lower because of time constraints that were not captured in this controlled setting.

## Conclusions

In this exploratory study, which included one synthetic and three authentic images, greater clinical experience was associated with reduced detection of the evaluated synthetic image, possibly reflecting an overreliance on heuristic pattern recognition. However, whether this pattern generalizes beyond the selected image requires further study. The inability of human experts and generative AI models to consistently distinguish real images from synthetic ones highlights an emerging epistemological challenge in medical imaging. Educational frameworks must be adapted to train clinicians to critically evaluate the biological plausibility of digital images and to encourage more rigorous visual analysis of digital medical data.

## Data Availability

The data supporting the findings of this study are available from the corresponding author upon reasonable request, subject to any applicable privacy/ethical restrictions.

## Abbreviations

AI: Artificial intelligence
LMM: Large multimodal model
